# Verbal fluency in elderly with and without hypertension and diabetes from the FIBRA study in Ermelino Matarazzo

**DOI:** 10.1590/1980-57642016dn11-040011

**Published:** 2017

**Authors:** Nathalia Lais Morelli, Meire Cachioni, Andrea Lopes, Samila Sathler Tavares Batistoni, Deusivania Vieira da Silva Falcão, Anita Liberalesso Neri, Monica Sanches Yassuda

**Affiliations:** 1 Gerontologia, Escola de Artes, Ciências e Humanidades, Universidade de São Paulo, São Paulo, São Paulo, Brazil.; 2 Gerontologia, Faculdade de Ciências Médicas, UNICAMP, Campinas, São Paulo, Brazil.

**Keywords:** older adults, cognition, verbal fluency, executive function, hypertension, diabetes, idoso, cognição, fluência verbal, função executiva, hipertensão, diabetes

## Abstract

**Background:**

There are few studies on the qualitative variables derived from the animal category verbal fluency test (VF), especially with data originating from low-income samples of community-based studies.

**Objective:**

To compare elderly with and without hypertension (HTN) and diabetes mellitus (DM) regarding the total number of animals spoken, number of categories, groups and category switches on the VF test.

**Methods:**

We used the database of the FIBRA (Frailty in Brazilian Elderly) community-based study. The variables number of Categories, Groups and Category Switches were created for each participant. The total sample (n = 384) was divided into groups of elderly who reported having HTN, DM, both HTN and DM, or neither of these conditions.

**Results:**

There were no significant differences between the groups with and without these chronic diseases for VF total score or for the qualitative variables.

**Conclusion:**

Among independent community-dwelling elderly, the qualitative variables derived from the VF animal category may not add information regarding the cognitive profile of elderly with chronic diseases. Total VF score and the qualitative variables Category, Group and Switching did not differentiate elderly with and without HTN and DM.

## RESUMO.

**Introdução:**

Existem poucos estudos sobre as variáveis qualitativas decorrentes do teste de fluência verbal (FV) categoria animais, em especial com dados provenientes de amostra populacional de países com baixa renda.

**Objetivo:**

Comparar idosos com e sem hipertensão (HAS) e diabetes mellitus (DM) quanto ao número total de animais falados, número de categorias, grupos e trocas de categoria no teste de FV.

**Métodos:**

Utilizou-se o banco de dados do estudo FIBRA (Fragilidade em Idosos Brasileiros) de base populacional em Ermelino Matarazzo. Foram criadas as variáveis Categoria, Grupo e Troca de Categoria para cada participante. A amostra total (n = 384) foi dividida entre aqueles idosos que relataram ter HAS, DM, ambas ou nenhuma destas condições.

**Resultados:**

Não houve diferença significativa entre os grupos com e sem estas doenças crônicas para o escore total da FV nem para as variáveis qualitativas.

**Conclusão:**

Entre idosos independentes residentes na comunidade, as variáveis qualitativas derivadas da FV animais podem não adicionar informações sobre o perfil cognitivo de idosos com condições crônicas. O escore total da FV e as variáveis qualitativas Categoria, Grupo e Troca de Categoria não diferenciaram idosos com e sem HAS e DM.

## INTRODUCTION

The aging of the world population and growing prevalence of cognitive impairments in the elderly has led to great interest in instruments that can detect cognitive changes beyond those expected in healthy aging. Early identification of elderly with mild cognitive impairment or dementia is important for planning and implementing care and treatment. For this purpose, the most widely-used instruments are the Mini-Mental State Exam, the Verbal Fluency (VF) test and the Clock Drawing Test.^1^ The VF animals category test entails participants producing names of animals for 60 seconds. Besides total score (number of animals produced), qualitative scores extracted from the test can be analyzed, such as number of animal categories cited, number of groups formed in the same category (sequence of three or more animals belonging to the same category) and number of category switches.^2^ The VF semantic test requires a search drawing on previous conceptual knowledge held, based on a given target category, e.g. animals or fruit. On the VF animals test, the individual may produce words such as “horse, pig, cow and sheep” in succession, owing to the close associations with the concept of farm animals.^3^ During the task, the individual is expected to mention different categories of animals, where such category switching can produce better performance on the test.

To exemplify, in a previous study,^4^ young adults produced more words and performed more category switches during the VF semantic tasks (supermarket items). However, results were similar across different age groups for size of the group of items within the same category. No gender differences were found for total words produced on any of the VF tasks, but men produced larger groups on both tasks while women performed more switches than men on the VF semantic task. The study showed that fewer group switches resulted in lower total words produced among adults that were older.

Category switching appears to rely on the integrity of the frontal lobes. These regions are important for initiating search processes in semantic networks and for flexibility in the use of cognitive strategies.^3^^,^ 5 The VF semantic task^6^ appears to depend on involvement of the left and right pre-frontal cortex, as well as the left temporal lobe.

Besides the effect of age, the VF test is also influenced by education. A previous study^7^ showed that elderly with high educational level performed better on total VF and also on the first 15-second interval, number of categories cited, and on number of category switches. Thus, scores derived from the test should be analyzed according to the sociodemographic characteristics of the individual.

The VF animals test is widely use in the diagnosis of diseases that affect cognition, e.g. dementias and their pre-clinical stages. In general, most studies use total score on the VF animals tests but few studies have investigated the contribution of the variables number of categories mentioned, number and size of groups, and category switching for identifying cognitive changes.

Some studies^8^^-^ 10 have suggested that hypertension (HTN) is a risk factor for cognitive decline. Cognitive dysfunction can result from long-term exposure to vascular risk factors, such as HTN. Uncontrolled HTN can affect attention, episodic memory, processing speed, and executive functions.^9^^-^ 10 A study investigating primary attention^10^ found worse performance on the VF animals test among hypertensive older adults, even when in use of drug treatment.

Additionally, other studies have highlighted that diabetes mellitus (DM) may also represent a risk factor for cognitive decline.^11^ A recent study based on data from the National Alzheimer's Coordinating Center's Uniform Data Set (NACC-UDS)^12^ identified elders with normal cognition (N = 7,663) and mild cognitive impairment (N = 4,114) and compared subjects with and without DM for cognitive composite score and domain specific sub-scores. The results from the database revealed a higher rate of diabetes among those with cognitive impairment and that diabetics had lower cognitive performance. However, diabetics did not have significantly more rapid cognitive decline on the longitudinal follow-up.

Few studies exist on clustering and switching variables of the VF animals test, especially with data derived from community samples of low-income countries. No previous studies comparing elderly with and without HTN and DM for qualitative variables from the VF animals test were found. Therefore, the objective of the present study was to compare elderly with and without the chronic diseases HTN and DM for total animals produced on the VF test, number of animal categories cited, number of groups formed by a sequence of two or more names belonging to the same category, and number of category switches, during the VF animals category test. The original hypothesis of the study was that the qualitative variables derived from the VF would differentiate the diagnostic groups and that these groups would have similar total scores on the VF test.

## METHODS

### Participants.

This study was carried out using the database of the FIBRA (Frailty in Brazilian Elderly) community-based study, whose objective was to determine the prevalence of frailty in elderly and associated variables. A total of 384 older adults took part in the present cross-sectional study.

Inclusion criteria were: being aged 65 years or older; and being a permanent resident of Ermelino Matarazzo.^13^ Exclusion criteria of the FIBRA study were: having a prior diagnosis of dementia or signs suggestive of dementia at the recruitment interview for the study; permanent or temporary walking disability, except with the use of a walking aid; localized loss of strength or stroke-induced aphasia; severe impairment of motricity, speech or affective disorders associated with advanced Parkinson's disease; severe auditory or visual impairments; and being in a terminal state.

The present study included all participants of the FIBRA study in Ermelino Matarazzo, i.e. participants scoring below the cut-off score on the Min-Mental State Exam were not excluded. This decision was owing to the need to explore the impact of the chronic diseases (HTN and DM) in a sample with greater variability in cognitive performance.^14^

### Instruments.

The instruments used for characterizing the sample profile were a sociodemographic questionnaire, the Mini-Mental State Exam (MMSE), and the Brief Cognitive Screening Battery (BCSB).^15^

The BCSB was developed to assess the cognition of individuals with low education and includes memorizing 10 black and white drawings, the Verbal Fluency (VF) test – animals category, and the Clock Drawing Test (CDT). In the memorizing task, individuals are asked to name the drawings and then recall the them incidentally (Incidental Memory). Subsequently, subjects examine the drawings for 30 seconds and attempt to recall them (Immediate Memory). Participants have another opportunity to study the drawings for 30 seconds and recall them (Learning). After performing both the VF and CDT tests, delayed recall (Delayed Memory) and recognition of target figures among 10 distractors is performed.

The CDT is an instrument for detecting cognitive changes. The test assesses visuoconstructional and visuospatial functions. In the present study, the CDT was scored based on Shulman's criteria.^16^

The presence or absence of HTN and DM was based on self-reports. The following questions from the FIBRA protocol were used: “Do you have hypertension?” “Are you diabetic?”. Positive responses for each of the diseases were considered and participants that answered yes to both questions were defined as individuals with HTN and DM. Blood pressure was measured three times with participants in a sitting position and twice in a standing position. An automatic pressure monitor was used (Omron Hem 705 CP). Average values for systolic blood pressure (SBP) and diastolic blood pressure (DBP) were then calculated.

### Procedures.

On the VF test, interviewees must cite examples, in this case, animals in one minute, divided into 15-second intervals. The test assesses vocabulary and lexical access, restricting the semantic search to a given category.

For the present study, the participants' responses on the VF animals test were reanalyzed to calculate number of categories, number of groups and number of group switches. Category was defined as a class of animals sharing a core characteristic distinguishing it from other classes of animals (e.g. domestic versus wild, aquatic versus land, flying versus crawling). The following categories were employed: domestic and farm animals, wild animals, insects, fish, birds and reptiles. Groups were defined as a sequence of two or more names of animals of the same category. Switching was defined as a change from the concept underlying a sequence of names the speaker was producing to another concept represented by a new sequence of names belonging to another category, or by single names not belonging to the category being produced (e.g. [duck, hen, horse], [dragonfly, mosquito], bear, butterfly, [tiger, lion, zebra], alligator, [shark, salmon, tuna].^2^^,^ 7

For the present study, 383 records from the FIBRA studies were reviewed. The variables Category, Group and Switches were created for each participant by two specially trained researchers. At the start of the process, for training purposes, both researchers assessed the same new variables for around 30 records and disparities were resolved through discussions. The remaining records were assessed independently by one of the researchers. After completing the calculation of the new scores, the results registered in the records were typed into the database of the FIBRA study. In the present study, the number of animals produced every 15 seconds of the test were used.

### Statistical analyses.

The presence of a normal distribution was assessed using the Kolmogorov-Smirnov test. ANOVA was used for the descriptive analyses comparing the sociodemographic characteristics of the groups with HTN, DM, HTN+DM and without either of these chronic diseases, given that age, income and education had a normal distribution. The Kruskal-Wallis test was employed to compare cognitive scores among the groups because the cognitive variables had a non-normal distribution. Correlation analyses were performed using Spearman's test.

## RESULTS

The study sample comprised 384 individuals. Mean age was of participants was 72.3 years (SD = 5.8) and 60.2% were women. The most prevalent chronic diseases were hypertension (59.8%), arthrosis (29.1%), diabetes (21.9%) and cardiac disease (17.7%) and 57.8% reported having 1 or 2 chronic diseases. The majority of participants had four years of education (60.5%) and most had a family income of 1-3 minimum wages per person (53.2%). Regarding cognition, 16.6% scored below the education-adjusted cutoff on the MMSE.

The sociodemographic characteristics of the sample for participants without HTN or DM (normal group), with HTN, with DM and with both diseases are given in Table 1. The groups studied were equivalent for age, education and income.

**Table 1:** Sociodemographic characteristics of sample grouped by participants with HTN, DM, HTN and DM, and individuals without either disease (Normal).

	Normal (n = 112)	HTN (n = 181)	DM (n = 22)	HTN+DM (n = 69)	p-value
Age	72.29 (5.72)	72.38 (5.59)	71.90 (4.73)	72.33 (6.65)	0.94
Education	3.83 (3.44)	3.87 (2.56)	3.00 (2.45)	2.94 (2.42)	0.39
Income (reais)	1301.50 (1368.03)	1119.15 (1093.88)	1656.45 (2135.57)	1259.59 (1405.34)	0.74

HTN: hypertension; DM: diabetes mellitus; M: mean; SD: standard deviation; p-value refers to Kruskal-Wallis test.

Comparison of cognitive performance revealed no significant differences among the groups (Table 2). Similarly, no group differences were observed for the variables Category, Group and Group Switches.

**Table 2:** Cognitive characteristics of sample grouped by participants with HTN, DM, HTN and DM, and individuals without either disease (Normal), expressed as mean (standard deviation).

	Normal (n = 112)	HTN (n = 181)	DM (n = 22)	HTN+DM (n = 69)	p-value
MMSE	24.23 (3.39)	23.68 (3.88)	23.64 (2.80)	23.01 (4.25)	0.30
CDT	2.47 (1.74)	2.47 (1.72)	2.32 (1.59)	3.94 (11.73)	0.94
DR-BCSB	7.21 (1.95)	7.58 (1.82)	7.00 (2.56)	8.85 (11.26)	0.10
VF Total	12.06 (3.42)	11.78 (3.60)	11.64 (3.06)	12.97 (11.08)	0.79
VF 0-15	4.97 (1.68)	5.09 (1.40)	3.14 (1.96)	4.20 (11.67)	0.71
VF 16-30	3.00 (1.41)	2.93 (1.46)	3.14 (1.96)	4.20 (11.76)	0.85
VF 31-45	2.23 (1.47)	2.05 (1.48)	1.86 (1.35)	3.52 (11.75)	0.64
VF 46-60	1.87 (1.34)	1.76 (1.65)	1.68 (1.32)	3.27 (11.80)	0.64
Category	3.20 (1.12)	2.98 (1.03)	2.91 (1.02)	2.93 (0.93)	0.27
Group	1.60 (0.84)	1.53 (0.90)	1.55 (0.85)	1.54 (0.76)	0.94
Switches	4.37 (2.45)	4.24 (2.57)	4.05 (2.38)	4.18 (2.47)	0.92

HTN: hypertension; DM: diabetes mellitus; MMSE: Mini-Mental State Examination; DR-BCSB: Brief Cognitive Screening Battery; DR: delayed recall; M: mean; SD: standard deviation; p-value refers to the Kruskal-Wallis test.

No significant correlation was evident among the VF variables Category, Group and Group Switches or for SBP and DBP readings (Table 3).

**Table 3:** Results of correlation analyses between SBP and DBP with cognitive variables investigated.

	VF Total	Category	Group	Group switching
Systolic pressure	0.038	0.009	0.076	0.029
Diastolic pressure	–0.045	0.036	–0.035	0.011

**Correlation significant at 0.01 level;

*Correlation significant at 0.05 level.

(N = 378). SBP: systolic blood pressure; DBP: diastolic blood pressure.

## DISCUSSION

The objective of this study was to assess the contribution of measurements derived from the VF animals (number of categories, groups and switches) in differentiating elderly with HTN, DM, HTN and DM from elderly without these conditions. The results showed that the qualitative variables did not add information about the groups. None of the VF variables differentiated between the groups with and without the clinical conditions. The qualitative variables were expected to reveal cognitive problems not detected by the overall VF score.

The results of the present study were similar to those of an earlier investigation^17^ which found no significant differences for cognitive variables between hypertensive and normotensive elderly users of a Basic Health Unit in the interior of São Paulo state. However, in the cited study, the difference for VF animals approached statistical significance, where this difference between groups would likely reach significance in a larger sample. The authors suggested that the absence of an effect of HTN on cognition might be explained by the fact that participants were undergoing treatment. Another study also revealed that the performance of elderly with HTN not in use of pharmacological treatment was lower than patients undergoing treatment.^18^ In the present study, it is plausible to deduce that the absence of differences in cognitive performance of the groups was associated with adherence to pharmacological treatment. In the sample, 96.4% of HTN participants reported undergoing treatment, compared with 85.7% of DM patients.

Studies^19^ have shown that elevated glycemia is associated with poorer cognitive function among elderly with DM and coronary heart disease. Disease duration and level of glycemic control also appeared to be determinants for the emergence of cognitive impairments. In the cited study, the presence of DM was associated with lower performance on executive functions, including VF. In the present study, this finding was not confirmed for VF or MMSE performance, corroborating the results of a previous study.^20^

Limitations of the present study include the fact that data on the clinical conditions were based on self-reports. Therefore, it was not possible to clinically confirm that the elderly had the diseases reported. In addition, subjects' low educational level may have affected understanding of the questions about health. Additionally, the vast majority of participants were undergoing pharmacological treatment for the clinical conditions investigated, precluding comparison of treated and untreated individuals to assess the influence of treatment on the results.

In conclusion, the present study comparing groups with clinical conditions (HTN, DM, HTN and DM, without HTN or DM) found that none of the cognitive variables differentiated these groups. These data suggest that qualitative variables do not add further information to total VF score. This study was performed in community-dwelling elderly and the value of these qualitative variables may be more evident in elderly with more severe cognitive impairment. The results obtained should be interpreted in the context of a population-based sample of low-educated elderly. Previous studies have tended to investigate elderly with 12 years or more of education.
